# Does Preoperative CT Improve Outcomes in Femoral Neck Fracture Fixation? A Retrospective Study

**DOI:** 10.3390/jcm14165917

**Published:** 2025-08-21

**Authors:** Ludovico Lucenti, Andrea Sodano, Flora Maria Chiara Panvini, Andrea Vescio, Giacomo Papotto, Gianluca Testa, Vito Pavone

**Affiliations:** 1 Department of General Surgery and Medical Surgical Specialties, Section of Orthopaedics, A.O.U. Policlinico Rodolico-San Marco, University of Catania, 95123 Catania, Italy; 2 Department of Precision Medicine in Medical, Surgical and Critical Care (Me.Pre.C.C.), University of Palermo, 90133 Palermo, Italy; 3 Department of Life Science, Health, and Health Professions, Link Campus University, 00165 Rome, Italy; 4 Department of Orthopaedic and Trauma Surgery, “Mater Domini” University Hospital, “Magna Græcia” University, 88100 Catanzaro, Italy; 5 Department of Orthopaedics and Traumatology, Emergency Hospital Cannizzaro, 95123 Catania, Italy

**Keywords:** computerized tomography, cannulated screw, hip, femoral fracture, femoral neck, subcapital fracture

## Abstract

**Background/Objectives**: Femoral neck fractures are a growing concern due to their increasing incidence in both elderly and younger populations. Preoperative CT scan evaluation is often used for better understanding of fracture patterns of femoral neck fractures that need to be treated using cannulated screws. **Methods**: The present study retrospectively analyzed 55 patients treated with cannulated screw fixation over seven years. Preoperative CT scans, fracture classification (Garden and Pauwels), and surgical timing were evaluated. **Results**: All 55 patients were treated with three cannulated screws by nine fellowship-trained surgeons. The average age of the CT-yes group was 54.44 years (SD 15.45), while the average age of the CT-no group was 56.93 (SD 14.95). Differences in age among the two groups were not statistically significant. In 4 patients, the treatment with cannulated screws failed, leading to a subsequent total hip arthroplasty for avascular necrosis (AVN). Three of them belonged to the CT-yes group, while only one belonged to the CT-no group. A statistical analysis showed no significant differences between patients who underwent a CT scan and those who did not have a CT scan and their results (*p* = 0.282). Results indicate no statistically significant difference in outcomes between patients who underwent a CT scan before the surgical treatment and those who did not, while a well-conducted X-ray assessment is essential and can be sufficient to fully understand and treat most of the fractures. **Conclusions**: The risks of avascular necrosis and non-union must be considered in the decision-making process regarding the suitable treatment. Early surgery did not significantly improve treatment results, but it is recommended. Fixation with cannulated screws remains a good treatment, especially for some patterns of fractures and younger patients. Given the study’s limitations, including the small sample size and retrospective nature, prospective multicenter studies are warranted to better understand the role of CT scans in optimizing surgical planning and improving patient outcomes.

## 1. Introduction

Hip fractures are very frequent injuries, with an incidence that has been rising significantly over the last decade [1]. They represent a significant public health concern, with a growing global burden [2]. In older people, these fractures are mainly related to low-energy injuries [3]. With the increase in the number of traffic accidents, the incidence of these fractures has doubled in recent years, and these fractures are occurring more, not only in the elderly but also in younger people [4]. The global incidence is expected to rise dramatically due to aging populations and urbanization. Predictably, by the year 2050, the worldwide annual incidence of hip fractures will increase to 6–8 million fractures [5]. This expected increase poses a significant challenge for healthcare systems across both high-income and low- to middle-income countries with a profound social and economic impact. This fracture implies a vital effect on families and society regarding resources and costs (related to hospitalization, surgery, rehabilitation and long-term care) on healthcare infrastructure and national economies [6]. Femoral neck fractures account for more than 50% of hip fractures, making them a critical focus for prevention and optimized treatment strategies. These are biomechanically complex due to the anatomy and load transmission across the hip joint.

**Figure 1:**
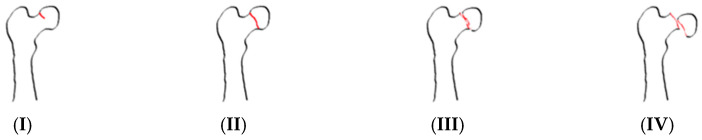
Garden Classification. (**I**): Incomplete stable fracture; (**II**): Complete undisplaced fracture; (**III**): Partially displaced fracture; (**IV**): Fully displaced fracture.

**Figure 2:**
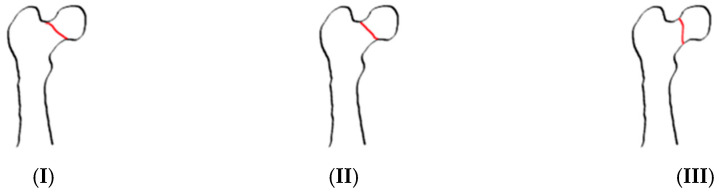
Pauwels Classification. (**I**): Fracture line <30° from horizontal; (**II**): Fracture line between 30° and 50° from horizontal; (**III**): Fracture line >50° from horizontal (most unstable).

The most used classifications are Garden and Pauwels. In the first one, ref. [7] femur neck fractures are classified by fracture displacement based on an AP radiogram into non-displaced (type I and II) and displaced (type III and IV) fractures. Garden type I describes an incomplete or impacted fracture, Garden type II a complete fracture without displacement, Garden type III a complete fracture with partial displacement, and Garden type IV a complete fracture with full displacement [8] (Figure 1). The Pauwels Classification [9] takes into account the biomechanical forces acting on the fracture. This classification emphasizes the angle of the fracture in relation to the horizontal axis, which directly correlates with biomechanical stability [10]. Type I describes a dominating compression force with a fracture line up to 30° to the horizontal plane. Shearing stress is present in type II but not predominant; the fracture line lies between 30° and 50°. In the third type, with a fracture line above 50°, shearing stress is predominant leading to fracture displacement. Vertical fractures are associated with high shear forces, reducing intrinsic fracture stability and increasing the risk of displacement and non-union unless adequate fixation is achieved (Figure 2).

Due to the peculiar vascular anatomy, these fractures often lead to avascular necrosis of the femoral head and non-unions. For this reason, treatment is often challenging. Many surgeries exist for femoral neck fractures in young adults, but they are usually treated with closed reduction and internal fixation (CRIF) using cannulated compression screw (CCS) [11,12]. Other options include dynamic hip screw, intramedullary nail, hemiarthroplasty and total hip arthroplasty [13,14].

CCS fixation aims to neutralize displacement forces through optimal placement. Biomechanical studies confirm that an inverted triangle configuration provides superior resistance to rotational and shear forces. The most crucial aspect is to achieve subchondral purchase in the femoral head while avoiding iatrogenic joint penetration. Inferior calcar support with one screw abutting the calcar femorale enhances mechanical stability and reduces the risk of varus collapse [15,16].

Furthermore, achieving an anatomic or slight valgus reduction reduces shear and promotes compressive loading across the fracture plane, which is more favorable for healing. Therefore, the interplay between fracture morphology, reduction quality, and screw configuration is critical to minimizing mechanical failure and optimizing outcomes.

This procedure has been primarily used and appreciated by surgeons because it is a pretty straightforward operation with limited risk of complications (like blood loss, nerve injury, or infections), especially when compared to a partial or total hip replacement (THR) [17,18]. Risk of other complications such as avascular necrosis is largely reported in the literature [19]. The latter is a very serious issue, and the possibility of managing complication is carefully considered in clinical practice before, during and after surgery [20]. Hip replacement has gained popularity when an acceptable reduction is not possible, and when the risk of avascular necrosis is very high, especially in the elderly. Nonetheless, indications are still disputed and vary primarily based on the patient’s characteristics. An appropriate reduction is considered achieved when proper anatomical alignment of the fractured femoral neck is visible on both anteroposterior and lateral X-ray views. It optimizes the possibility of healing minimizing complications such as non-union or avascular necrosis [21].

Rapid surgical treatment is preferred, specifically in the absence of contraindications [14].

In 2017, a new device called Femoral neck system (FNS) (DePuy Synthes, Johnson & Johnson) was introduced for the internal fixation of femoral neck fractures [18,22]. The latter has obtained excellent results so far, with a better union time, operation time, and functionality when compared to the CCS fixation, but with a higher blood loss and no difference in hospital stay and femoral neck shortening [23]. It offers faster union times and fewer reoperations in Pauwels III fractures, its benefits over CCS are not significant in less vertical or undisplaced fractures [24]. The FNS integrates features of both CCS and DHS, combining a minimally invasive technique with angular stability and controlled impaction [25].

Nonetheless, CCS fixation remains the most used treatment for femoral neck fractures, especially in young individuals. This operation consists in fixing the femoral neck fracture with a few screws (usually 3). Using a single small approach or a few centimetric accesses, multiple cannulated screws are implanted into the femoral neck in a parallel way. To reach the correct position of the screws, some guide wires are previously inserted through X-ray guidance, and they can easily be adjusted if needed. Quick and precise positioning of the guide wires is fundamental. Afterward, the cannulated screws are inserted on the previously implanted guide wires; a washer can be applied [26].

Outcomes of these fractures seem to be very variable, depending on many factors such as patients’ characteristics, the type of fracture, and the position of the screws. Vertical fractures, displaced fractures and unstable patterns have the highest risk of nonunion. Achieving a good reduction can be essential for the healing. Tobacco use, alcohol abuse, low bone density, and impaired vascularization can delay or interfere the bone healing [27,28].

CCS can be removed 1 or 2 years after the implantation in young and middle-aged patients. This leaves screw tunnels in the bone, which could represent a locus minoris resistentiae and lead to a re-fracture [29]. A study analyzed the effects of screw tunnels on proximal femur strength [30], and it found that bone which developed around the screws was more similar to the cortical bone than the cancellous bone, according to Wolff’s law. Other studies identified the calcar under the femoral neck as the most stressed area, and this could explain why if the medial femoral cortex is not properly aligned or there is a bone defect, internal fixation fracture, or hip varus deformity could occur after the operation. An inverse correlation was found between screw tunnel diameter and the compressive strength of the single screw tunnel, so a smaller diameter of the tunnel was associated with higher compressive strength of the proximal femur. Finally, they demonstrated that the inverted triangle shape was the best one in terms of compressive strength, but in every case, the proximal femur with tunnel resulted in less compressive strength than the normal ones. Nonetheless, they also demonstrated that the proximal femur with tunnels can withstand day-to-day stress with no other intervention, seeing that it could afford at least 4.6 times the weight of the body in static conditions.

Despite the evolving landscape, CCS remains preferred for its simplicity, low morbidity, and efficacy in selected fracture types. The choice of fixation must therefore be individualized, balancing stability requirements against surgical invasiveness, patient factors, and available resources [31].

Furthermore, an important topic is the timing between trauma and surgery. The literature on this is rich but needs to be clarified. Many studies highlight the importance of early surgery to restore the vascularization of the femoral head as soon as possible [32], while others show no significant difference with delayed surgery [33].

Amid this debate, less attention has been given to the role of detailed preoperative imaging (specifically CT scans) in influencing surgical outcomes.

Adequate preoperative surgical planning and accurate comprehension of the lesion using second-level diagnostic imaging CT scan have not been investigated as a possible key factor in reaching optimal outcomes in the treatment of fractures [34].

Despite being increasingly used in orthopaedic practice for complex fracture assessment, CT imaging remains under-investigated in the context of femoral neck fractures, particularly in relation to treatment success, probably for its habitually use for articular fractures and easy access to it.

The aim of this study is to evaluate the correlation between the success of the treatment with cannulated screws for femoral neck fractures and the preoperative evaluation through a CT scan.

## 2. Materials and Methods

### 2.1. Study Design: Retrospective Cohort Study

A retrospective review was performed of all patients who had undergone surgical treatment with cannulated screws fixation for a femoral neck fracture over a 7-year period at a single institution between January 2016 and December 2022. All surgeries followed the same standardized protocol in terms of pre- intra- and post-operative assessment.

Primary demographic information was collected, including laterality, sex, and age.

All skeletally mature patients with a femoral neck fracture undergoing surgical fixation with cannulated screws were included. Exclusion criteria were patients with fractures different than the femoral neck, pathological fractures, skeletally immature patients, patients with inadequate radiographs, and patients with less than 1 year of follow-up.

Pre-and post-operative radiographs were examined to determine the type of fracture, the correct indication for fixation, and the position of the screws. The correct indications for screw fixation were undisplaced, minimally displaced femoral neck fractures (Garden I–II) or displaced fractures (Garden III–IV) after anatomical reduction in young active patients (<60 years) with high functional demands and good bone quality.

The optimal screw position was referred to the three-screw parallel configuration: screws parallel to each other, engaging the subchondral bone of the femoral head and widely spread within the femoral neck to maximize fixation in all planes. The “Inverted Triangle” pattern is the most commonly used with an inferior screw just above the calcar and two superior screws (in anterosuperior and posterosuperior positions) [35,36,37,38].

Two study authors, traumatologists with more than five years of experience, conducted all measurements (L.L. and G.T.). All radiographs were assessed blindly. Any discrepancies were discussed with the senior investigator (V.P.) before the final decision to consider the fracture properly healed or not was made.

There were 63 femoral neck fractures treated with cannulated screws during the study period. Eight patients were excluded from the study due to inadequate follow-up or radiographs. The last follow-up was determined by reviewing the patient charts and the last X-ray.

Fifty-five patients (35 women and 20 men) met all the inclusion criteria. The mean age of these patients was 55.71 (SD 15.11), and the mean follow-up time was 31.36 months (range: 14–42 months).

**Figure 3:**
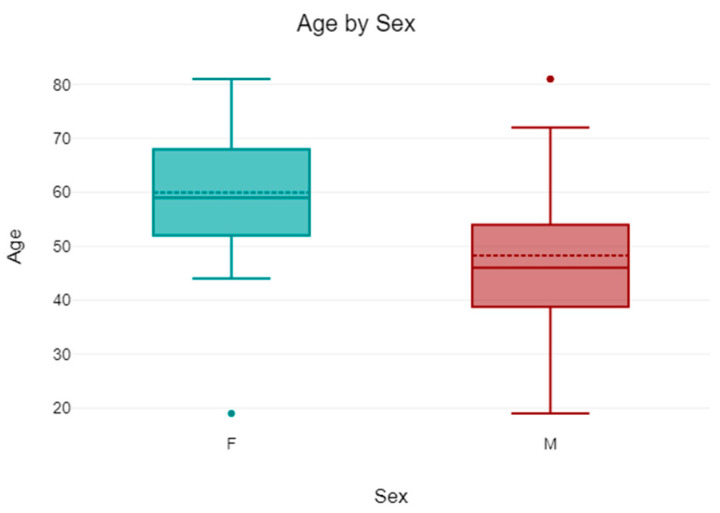
The scheme shows the different age ranges between males and females.

The mean age of the 35 women was 59.94 (SD 12.95), while the mean age of the 20 patients was 48.3 (SD 16.07) (Figure 3).

Of the 55 patients, 27 (49.09%) had a preoperative evaluation with a CT scan (CT-yes), while 28 (50.91%) patients did not undergo a CT scan evaluation (CT-no). The indication for CT scanning was determined at the discretion of the treating surgeons on a case-by-case basis.

**Figure 4:**
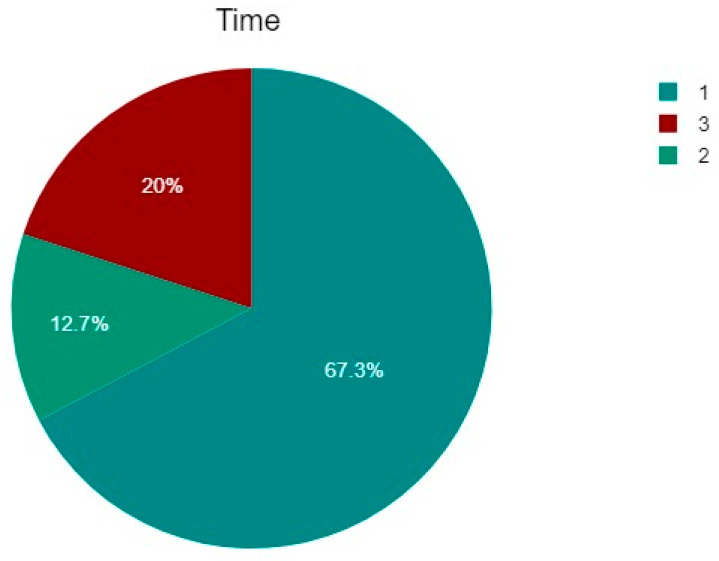
The percentage of the three groups: Group 1 (37 patients—67.27%) treated within 24 h from the diagnosis, Group 2 (7 patients—12.73%) treated within 48 h from the diagnosis, and Group 3 (11 patients—20%) treated after 48 h from diagnosis.

The timing of surgery was collected, distinguishing between surgery in the first 24 h (Group 1), 48 h (Group 2), or later than 48 h from diagnosis (Group 3). A total of 37 (67.27%) patients belonged to the first group, 7 (12.73%) to the second, and 11 (20%) to the third group (Figure 4). Surgical timing was established by the treating surgeon according to the patient’s general condition, the extent of the fracture, and the availability of the operating room and a dedicated team.

The fractures were also classified in according to Garden and Pauwels classifications.

**Figure 5:**
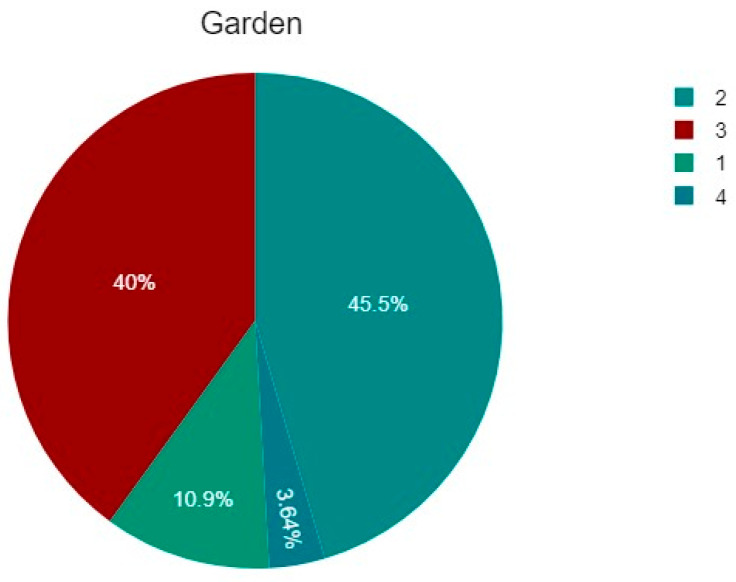
The percentage of fractures according to the Garden classification: type 1, 6 patients (10.91%), type 2, 25 (45.45%), type 3, 22 (40%), and type 4, 2 patients (3.64%).

Regarding Garden classification, of the 55 patients, 6 (10.91%) were type 1, 25 (45.45%) were type 2, 22 (40%) were type 3, and 2 (3.64%) were type 4 (Table 1 and Figure 5).

**Table 1:** Number and percentage of fractures according to the Garden Classification and Pauwels Classification.

	Garden Classification									
	1		2		3		4		Total	
	*n*	%	*n*	%	*n*	%	*n*	%	*n*	%
Total	6	10.91%	25	45.45%	22	40%	2	3.64%	55	100%
	Pauwels Classification									
	1			2			3		Total	
	*n*	%		*n*	%		*n*	%	*n*	%
Total	11	20%		27	49.09%		17	30.91%	55	100%

**Figure 6:**
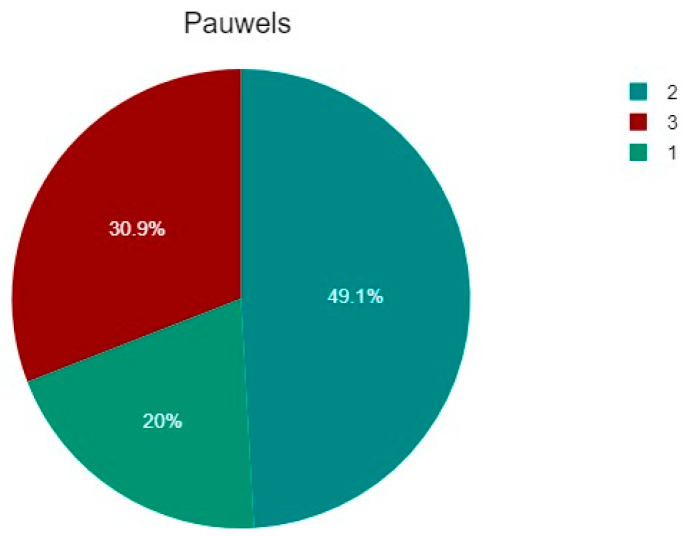
The percentage of fractures according to the Pauwels classification: type 1, 11 patients (20%), type 2, 27 patients (49.09%), and type 3, 17 patients (30.91%).

About Pauwels classification, of the 55 patients, 11 (20%) were type 1, 27 (49.09%) were type 2, and 17 (30.91%) were type 3 (Table 1 and Figure 6).

Table 2 below shows the percentages of fractures according to both Pauwels and Garden Classification.

The treatment was considered successful (success) if no more surgery was needed until the last follow-up. In the case of surgical treatment afterward, it was considered a failure of the previous treatment (fail) with cannulated screws. Treatment failure was defined as the need for subsequent surgical intervention (mainly conversion to total hip arthroplasty) due to complications such as avascular necrosis, non-union, or loss of fixation. Radiographic evidence of fracture non-union, femoral head collapse, screw back-out or migration, and persistent pain with functional limitation were also considered indicative of failure.

### 2.2. Surgical Technique

All the 55 patients were treated with three cannulated screws by nine fellowship trained surgeons. No main technical differences between surgical procedures were verified.

Eventual antithrombotic prophylaxis with LMWH was suspended 10–12 h before surgery. Antibiotic prophylaxis was performed between 1 h and 30 min before surgery with Cefazolin 2 g or with another antibiotic class when contraindicated. Spinal anesthesia was the first choice. When our anaesthesiologist equipe encountered contraindications, general anesthesia was performed.

The patient was positioned supine on a fracture table with a traction device to obtain the best reduction when needed. The contralateral leg was placed in a leg holder in flexion to obtain adequate lateral views of the hip, considering that the C-arm image intensifier was used during all phases of surgery. The C-arm was positioned contralateral to the surgeons.

In all cases reported in this work, a satisfactory close reduction was achieved using the classical longitudinal traction maneuver and internal rotation of the extremity checking intra/extra rotation under image intensifier control in 2 projections: AP and Lateral view [39].

A small lateral incision was used. Guide wires were inserted freehand under image intensifier control, in some cases (in according to the preferences of the first surgeon) with an aiming device to make them parallel to the femoral neck axis and each other. The chosen screws layout was the inverted triangle shape, with a proximal base and distal apex. The first wire inserted was the inferior one positioned in the calcar region to provide strong cortical support; afterwards, the superior wires were inserted one more anteriorly and one more posteriorly according to the preference of the orthopedic surgeon.

After determining the correct screw length, a 5.0 mm cannulated drill bit was used to prepare screw insertion, and three 6.5 mm cannulated screws were positioned. When traction was used, it was released before final screw tightening.

Final image intensifier views were obtained.

Antithrombotic prophylaxis with LMWH was restarted 8–10 h after surgery.

Post-operatively active and passive exercises in bed were permitted. Weight-bearing was not allowed for six weeks.

Weekly follow-up was performed for wound healing and radiographic follow-up at 1, 3, 6, and 12 months.

## 3. Results

All the 55 patients were treated with three cannulated screws by nine fellowship trained surgeons. The average age of the CT-yes group was 54.44 years (SD 15.45), while the average age of the CT-no group was 56.93 (SD 14.95). Differences in age among the two groups were not statistically significant. In 4 patients, the treatment with cannulated screws failed, leading to a subsequent total hip arthroplasty for avascular necrosis (AVN). Three of them belonged to the CT-yes group, while only one belonged to the CT-no group (Table 3 and Figure 6). No cases of infections or wound issues were observed. The failure rate was 11.11% (95% CI: 2.9–29.1%) in the CT group and 3.57% (95% CI: 0.6–17.7%) in the non-CT group. A statistical analysis showed no significant differences between patients who underwent a CT scan and those who did not have a CT scan and their results (*p* = 0.282). Statistical analysis was performed using IBM SPSS Statistics for Windows, version 27 (IBM Corp., Armonk, NY, USA).

A possible relation between the timing of the operation and a good result was also investigated. Patients operated on within the first 24 h belonged to Group 1, those operated on within the first 48 h belonged to Group 2, and those operated on after 48 h belonged to Group 3 (Table 4). A total of 37 (67.27%) patients belonged to the first group, 7 (12.73%) to the second, and 11 (20%) to the third group.

**Figure 7:**
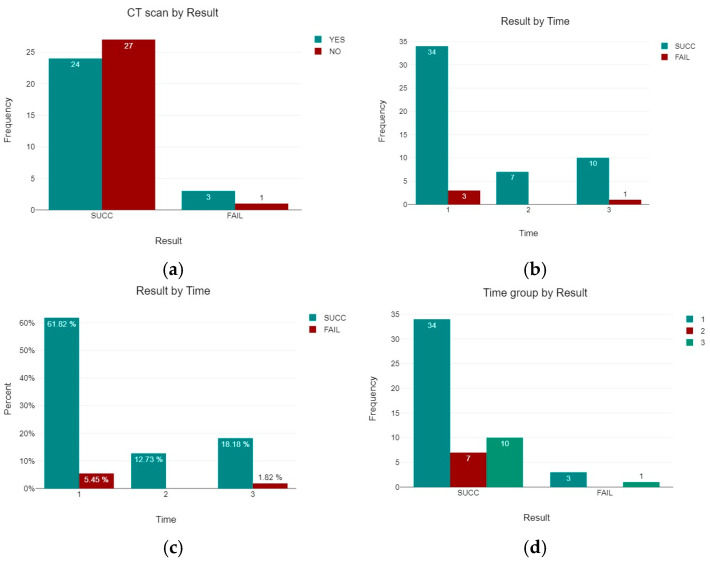
(**a**) Number of successful results and number of failures in patients who underwent a CT scan and in those who did not have a CT scan. (**b**) Number and (**c**) percentage of patients for each time group and successful results. (**d**) Result for each time group. Success was defined as healing of the fracture, while failure was defined as the need for subsequent surgical intervention (mainly conversion to total hip arthroplasty) due to complications such as avascular necrosis, non-union, or loss of fixation, radiographic evidence of fracture non-union, femoral head collapse, screw back-out or migration, and persistent pain with functional limitation.

The number and percentage of the patients in the three different groups and the percentage of successful results and failures are reported in Table 5 and Figure 7. A statistical analysis showed no significant differences between patients of the three groups and the percentage of success of the treatment (*p* = 0.726).

Table 6 shows the number and percentage of success and fail by Garden and Pauwels classification.

## 4. Discussion

The present study aimed to estimate how the execution of a preoperative CT scan can impact the result of a CRIF using CCS for femoral neck fractures. Although the frequency of femoral neck fractures is constantly increasing due to aging populations and traffic accidents, optimal treatment strategies are still discussed, especially concerning preoperative evaluation and the timing of surgery.

Currently, many classification methods for femoral neck fractures are based on standard X-ray imaging. Nonetheless, patients with femoral neck fractures present pain and the affected limb flexion and external rotation deformity. For these reasons, it’s difficult to take a standard anteroposterior (AP) radiograph of the pelvis or AP and lateral radiograph of the hip joint before surgery in order to plan it as best as possible [40].

Conversely, CT scan examination is without position limitation, and it allows a better understanding of the fracture [41]. Most of the studies presented in the literature regarding comparison between X-ray and CT-scan (other than MRI) are inherent occult fractures. Alabousi et al. [42] in a meta-analysis involving four studies and 418 patients have reported that CT has excellent specificity and moderate sensitivity for detecting hip fragility fractures after negative radiographs. It can be considered a second-line imaging modality. Nonetheless, MRI remains the gold standard, especially when CT is negative but clinical suspicion is high. Similar conclusions were reported by Kellock et al. [43] in their meta-analysis conducted on 13 studies with a total of 1248 patients. The authors have reported that CT is a reliable diagnostic tool for ruling in or out occult proximal femoral fractures in patients with negative X-rays and persistent symptoms, but MRI should be pursued if doubt remains after a negative CT.

Considering that CT scan offers an excellent fracture vision, compared to standard radiographs, its value in preoperative evaluation was examined. No statistically significant difference was found in surgical outcomes between patients who underwent a CT scan and those who did not (*p* > 0.05). These results are not consistent with the study by Liu et al. [44], stating that CT scans can afford comprehensive fracture knowledge. Nonetheless, their routine use does not substantially change the clinical outcomes when compared to well-conducted standard X-rays. Femoral neck fractures are still challenging to treat because of the risk of complications such as avascular necrosis, non-union, and neck shortening. Indications to different types of surgery are debated, but CRIF with CCS is the most used procedure worldwide, especially among young adults. Anatomical reduction and stable internal fixation are mandatory to minimize the complications [18]. The ideal surgical procedure also needs to be quick, with limited blood loss, short hospital stay, and cheap. It also needs to lead to an early mobilization of the patient, which is very important to reduce many complications such as bedsores, pulmonary and urinary tract pathologies, and deep vein thrombosis.

Also for these reasons, the timing of surgery for femoral neck fractures is another factor primarily evaluated in the literature, and early intervention is often recommended to restore vascularisation and decrease complications. The present study categorized patients based on surgical timing within 24 h, 48 h, and beyond 48 h after the diagnosis. Surgical timing was established by the treating surgeon according to many factors such as the patient’s general condition, the extent of the fracture, and the availability of the operating room and a dedicated team. General medical condition of the patient and its compliance to the treatment and the rehabilitation can influence the outcomes. Consistently with some other studies [1,32,45], the authors did not find significant differences in success rates across these groups (*p* = 0.726), suggesting that early surgery is recommended, but the exact window time may not be as significant as previously believed [46]. Gumustas et al. suggest surgical intervention when optimum conditions (such as expert, fresh and dedicated team and enough time to achieve anatomical reduction) are provided instead of emergent surgery after trauma [33].

This study utilized the two most used classifications to categorize these types of fractures: the Pauwels and Garden classifications. The classifications help predict biomechanical stability, risk of displacement, complications, and decision about treatment. Undisplaced fractures are usually treated with internal fixation, while displaced fractures are frequently treated with arthroplasty, due to higher displacement and risk of vascular necrosis [8]. In fact, AAOS suggests internal fixation only for type I-II according to the Garden classification, reserving arthroplasty surgery for stage III-IV fractures due to a greater risk of vascular interruption [8,47].

Osteosynthesis with cannulated screw is a less invasive option in comparison with hemi- or total hip arthroplasty, but this surgery is not without risk. The most described complications are avascular necrosis of the femoral head, non-union at the fracture site, and screw loosening, and they usually lead to a reintervention.

Also, Pauwel’s classification has been correlated to a greater risk of avascular necrosis of the femoral head, with some studies proposing Pauwel’s stage III as a risk factor. Nonetheless, there is no consensus in the literature, with some studies showing promising results in this kind of fracture when an excellent reduction is reached [48].

It is described in literature how obesity and smoking tobacco affect fracture healing, so the patient’s lifestyle could also be an adverse prognostic factor [49,50].

A recent study showed how age, comorbidities, and internal fixation implant removal are independent risk factors for avascular necrosis of the femoral head [51]. All these aspects can influence the results of the femur fixation.

Screws configuration was also explored and the most used is the inverted triangle shape, with the distal-most screw that has its entry point not distal to lesser trochanter, in order to avoid subtrochanteric fractures [52].

Healthcare resource utilization for hip fractures represents a significant economic burden, projected to rise with aging populations [53]. Femoral neck fractures, particularly when managed surgically, contribute heavily to this cost due to implant expenses, operative time, hospital stay, and rehabilitation [54,55].

While preoperative CT scans offer detailed imaging, their routine use must be justified from a cost-effectiveness perspective. In scenarios where plain radiographs provide sufficient diagnostic clarity, CT may not add meaningful value, as reflected in this study’s findings. Additionally, radiation exposure can be significant when considered across large patient populations [56].

Beyond its potential role in outcome prediction, CT imaging may hold clinical value in specific scenarios [57]. In equivocal cases or in patients with complex or comminuted fracture patterns, or in those with atypical femoral anatomy (e.g., due to prior surgery, congenital variation, or deformity), CT scans can aid in detailed preoperative planning [58]. CT scan could potentially prevent reoperation by improving preoperative planning, making its use selectively justifiable. The refined 3D visualization allows for more accurate assessment of fragment displacement, bone stock, and implant trajectory. Therefore, while routine use may not be warranted in all cases, selective use of CT may still play a crucial role in optimizing surgical strategy in certain subgroups [59].

Rehabilitation after surgery plays an important role in influencing short and long-term outcomes after femoral neck fracture fixation [60]. Early mobilization reduces many complications such as deep vein thrombosis (DVT), pressure ulcers, and pneumonia, especially in elderly populations. Nonetheless, in patients treated with CCS, weight-bearing is typically restricted for 6–8 weeks, delaying full ambulation [61]. Functional outcomes should ideally be evaluated with standardized scoring systems such as the Harris Hip Score (HHS) or EQ-5D to assess pain, range of motion, and quality of life. Unfortunately, many studies (including this one) do not include these metrics. These systems are critical for capturing patient-centered outcomes. Developing protocols emphasize early supervised physiotherapy to enhance healing and function. Tele-rehabilitation platforms and wearable sensors are also being trialed to improve compliance and monitor gait postoperatively. Future research should incorporate functional endpoints alongside radiological union to offer a comprehensive view of treatment success.

The present study has some limitations. One of the most important limitations regards the small sample size, in fact only 55 patients met the inclusion criteria. Interobserver reliability between the two traumatologists who evaluated all the X-rays was not performed, but any discrepancies were discussed with the senior investigator before the final decision was made. Another limitation is related to the retrospective nature of the study and to the biases typical of this kind of studies: the type of data collection, the accuracy of historical records, the ability to control for all potential confounding variables, such as patient compliance, variations in post-operative care, and differences in rehabilitation protocols. Furthermore, the applicability of the findings to other realities can be limited due to the evaluation of a single institution. Different procedures and protocols may vary significantly between different hospitals and for this reason, the results reported might not be appropriate to other contexts. Additionally, the treatment was judged successful or not based only on additional surgical intervention. In the examined cohort, only a small number of failure events were reported. This limited the power of this study. Even if this is a critical outcome, it does not fully describe the clinical results. Other important factors such as pain levels, functional mobility, quality of life, and patient satisfaction are not reported. The CT scan can potentially influence not only the healing but also some or all these aspects. Similarly, characteristics that may influence the percentage of healing such as obesity, tobacco use and patient’s lifestyle were not investigated.

The study did not account for differences in surgical expertise, surgical techniques, and subgroups of patients which can significantly influence the outcomes. The mean follow-up period was 31.36 months (14–42 months). Longer follow-up is useful to have more comprehensive knowledge about long-term outcomes such as late-onset avascular necrosis and osteoarthritis. Prospective randomized studies with a larger cohort are recommended to clearly evaluate the efficacy of preoperative CT scans and optimal surgical timing.

## 5. Conclusions

The present study suggests that preoperative CT scan does not significantly improve surgical outcomes compared to standard radiographs in CRIF using CCS for femoral neck fractures. In clinical practice, the decision to utilize a CT scan or other advanced imaging should be tailored to individual cases. When standard radiographs are unsatisfying or for complex fractures, it may be required and very helpful. Selective rather than routine use of CT may help optimize resource utilization without compromising outcomes.

Early surgical intervention remains advisable. It may not independently predict better outcomes when compared with timely X-ray-based planning. Nonetheless, according to the findings reported in the present study, timing alone is not the most decisive factor of success. The most important aspects are anatomical reduction and stable fixation.

Given the study’s limitations, prospective, multicenter, randomized controlled trials are necessary to validate the findings reported in the present study and clarify the role of preoperative imaging in improving long-term clinical outcomes. Indications for the use of CT scan should be reported in appropriate guidelines, especially for ambiguous fracture patterns. Future research should also incorporate functional scores and quality-of-life metrics to offer a more comprehensive evaluation of treatment success from both surgical and patient-centered perspectives.

## Figures and Tables

**Table 2:** Number and percentage of fractures according to both Pauwels and Garden Classification.

Garden	Pauwels							
	1		2		3		Total	
	*n*	%	*n*	%	*n*	%	*n*	%
1	3	5.45%	2	3.64%	1	1.82%	6	10.91%
2	6	10.91%	10	18.18%	9	16.36%	25	45.45%
3	2	3.64%	15	27.27%	5	9.09%	22	40%
4	0	0%	0	0%	2	3.64%	2	3.64%
Total	11	20%	27	49.09%	17	30.91%	55	100%

**Table 3:** Number of successful results after surgical treatment in patients who underwent a CT scan and in those who did not have a CT scan.

CT Scan Group	Total (*n*)	Success (*n*)	Failure (*n*)	Failure Rate (%)	95% CI
CT Performed	27	24	3	11.1%	3.9–28.1%
No CT	28	27	1	3.6%	0.6–17.7%
Total	55	51	4	7.3%	2.9–17.3%

**Table 4:** Number of patients for each time group and fracture classification.

Classification/Type	Group Delay 1 (<24 h)	Group Delay 2 (<48 h)	Group Delay 3 (>48 h)
Garden 1	2	0	3
Garden 2	15	3	6
Garden 3	17	3	1
Garden 4	2	0	0
Pauwels 1	6	1	3
Pauwels 2	17	4	5
Pauwels 3	13	1	2

**Table 5:** Number and percentage of patients for each time group and percentage of successful results. The statistical analysis showed no significant differences between patients of the three groups and the percentage of success of the treatment (*p* = 0.726).

Result	Time Group													
	1 (≤24 h)				2 (≤48 h)				3 (>48 h)				Total	
	*n*	%	% Within Time Group	% Within Result	*n*	%	% Within Time Group	% Within Result	*n*	%	% Within Time Group	% Within Result	*n*	%
SUCC	34	61.82%	91.89%	66.67%	7	12.73%	100%	13.73%	10	18.18%	90.91%	19.61%	51	92.73%
FAIL	3	5.45%	8.11%	75%	0	0%	0%	0%	1	1.82%	9.09%	25%	4	7.27%
Total	37	67.27%	100%		7	12.73%	100%		11	20%	100%		55	100%

**Table 6:** Number and percentage of successful results by Garden Classification and Pauwels Classification.

Garden	Result									
	SUCC				FAIL				Total	
	*n*	%	% Within Result	% Within Garden	*n*	%	% Within Result	% Within Garden	*n*	%
1	6	10.91%	11.76%	100%	0	0%	0%	0%	6	10.91%
2	23	41.82%	45.1%	92%	2	3.64%	50%	8%	25	45.45%
3	20	36.36%	39.22%	90.91%	2	3.64%	50%	9.09%	22	40%
4	2	3.64%	3.92%	100%	0	0%	0%	0%	2	3.64%
Total	51	92.73%	100%		4	7.27%	100%		55	100%
Pauwels	Result									
	SUCC				FAIL				Total	
	*n*	%	% within Result	% within Pauwels	*n*	%	% within Result	% within Pauwels	*n*	%
1	11	20%	21.57%	100%	0	0%	0%	0%	11	20%
2	25	45.45%	49.02%	92.59%	2	3.64%	50%	7.41%	27	49.09%
3	15	27.27%	29.41%	88.24%	2	3.64%	50%	11.76%	17	30.91%
Total	51	92.73%	100%		4	7.27%	100%		55	100%

## Data Availability

The data are available in the tables in this manuscript. Other data used to support the findings of this study are available from the corresponding author upon request.
